# Evaluating Night-Eating Syndrome in bariatric surgery patients: A cross-sectional study

**DOI:** 10.1186/s13104-024-06774-z

**Published:** 2024-04-25

**Authors:** Sarah Almuammar, Elham Aljaaly, Lama Alqarni, Hadeel Alazwari, Ruba Alsubhi, Nouf Alatawi

**Affiliations:** 1 Family Medicine Department, Faculty of Medicine, King Abdulaziz University, Jeddah, Saudi Arabia; 2 Clinical Nutrition Department, Faculty of Applied Medical Sciences, King Abdulaziz University, Jeddah, Saudi Arabia; 3 Faculty of Medicine, King Abdulaziz University, Jeddah, Saudi Arabia

**Keywords:** Night-eating syndrome, Bariatric surgery, Eating disorders, Public health

## Abstract

**Background:**

Night-Eating Syndrome (NES) is a complex eating disorder that has gained recognition in the context of bariatric surgery. However, its prevalence and associated factors in this specific patient population remain understudied, particularly in the Saudi Arabian context.

**Methods:**

This cross-sectional study was conducted at King Abdulaziz University Hospital in Jeddah, Saudi Arabia, from June to November 2022. Adult patients who had undergone bariatric surgery with a postoperative follow-up of 6 months were included. NES was assessed using the Night Eating Questionnaire (NEQ).

**Results:**

A total of 154 patients were enrolled in the study, with a mean age of 38.8 ± 11.4 years. The mean BMI before surgery was 44.8 ± 8.2 kg/m^2^, which reduced to 28.9 ± 5.8 kg/m^2^ post-surgery. Of these, 52 patients (33.8%) met the criteria for NES based on NEQ scores. The prevalence of NES was significantly higher among female patients, with 35 out of 83 females (42.2%) compared to 17 out of 71 males (23.9%) experiencing NES. While NES was not significantly associated with age, nationality, diet adherence, BMI, or surgery type, chronic diseases—particularly diabetes—emerged as significant risk factors for NES in post-bariatric surgery patients.

**Conclusion:**

NES is a prevalent concern among post-bariatric surgery patients, with distinct associations with gender and chronic diseases, particularly diabetes. This study provides valuable insights into NES prevalence and its risk factors in the Saudi Arabian context, highlighting the importance of addressing eating disorders within the framework of bariatric surgery care.

## Background

The prevalence of bariatric surgery has seen a steady increase impacting the lives of over 14.6 million individuals [1]. For patients with class II and III obesity, bariatric surgery represents a transformative journey, leading to significant weight loss and substantial improvements in physical health [2]. Notable one-year post-surgery outcomes include reduced waist circumference, lower glucose and insulin levels, decreased free fatty acids and triglycerides, and a modest increase in high-density lipoprotein levels [3]. However, this path to weight reduction often intersects with the challenges posed by eating disorders.

A formal diagnosis of eating disorders has been linked to post-bariatric surgery patients, some resorting to spontaneous or forced vomiting as a means of weight control [4]. Cravings, more prevalent in this group than in healthy-weight controls, add another layer of complexity [5]. Furthermore, night-eating disorders have surfaced as a noteworthy concern post-surgery, with patients describing symptoms persisting for up to eight years following the procedure [6]. The foundational work of Stunkard et al. [7] introduced the concept of Night-Eating Syndrome (NES), characterized by minimal breakfast intake, consumption of over 25% of daily calories after dinner, and disrupted sleep patterns. Mood disorders, poor sleep quality, metabolic syndromes, obesity, and eating disorders are among the factors associated with NES [8]. To diagnose NES, specific criteria must be met, including the consumption of at least 25% of daily intake after dinner and nighttime awakenings occurring at least twice a week for a minimum of three months [9]. NES has been found to affect 1.5% of the general population in the United States and 1.5% of adult Omani residents [10, 11].

Recent studies highlight the challenge of NES in post-bariatric patients, with an observed increase in NES behaviors two to three years post-surgery [12–15]. A retrospective analysis in Bahrain linked eating disorder behaviors with diminished postoperative weight outcomes, emphasizing the importance of addressing these behaviors [16]. Variability in NES prevalence has been documented, ranging from 1.9 to 8.9% depending on the diagnostic criteria, which underscores the complexity of diagnosing and managing NES [17]. While these studies underscore the importance of understanding nighttime eating habits and their impact on the outcomes of weight-loss therapy, only a limited number of investigations have been conducted in Saudi Arabia, examining the relationship between NES and postoperative prognosis.

The present study aims to bridge this knowledge gap by investigating the prevalence and risk factors associated with NES in patients who underwent bariatric surgery at King Abdulaziz University Hospital in Saudi Arabia.

## Methods

### Study design

This cross-sectional study employed a self-administered, web-based survey through Google Forms. The study was conducted in collaboration with the Family Medicine Department and the Obesity Clinic at the Medical Nutrition Unit of King Abdulaziz University Hospital in Jeddah, Saudi Arabia, from June to November 2022.

### Study participants

The study included adult men and women who had undergone bariatric surgery, were 18 years of age or older, and had a postoperative follow-up period of 1 year. Pregnant women in the postoperative phase were excluded from the study.

### Study instrument

The survey encompassed thorough inquiries into the following areas:

1. **Demographics and clinical history**: Participants were requested to provide details regarding their age, gender, and nationality. Furthermore, they were asked about any chronic medical conditions, including hypertension, dyslipidemia, and diabetes mellitus type 2.
2. **Anthropometric measurements**: Respondents were required to report their height in centimeters (cm) and their weight in kilograms (kg), both before the bariatric procedure and one-year post-procedure.
3. **Type of bariatric procedure**: Information was collected on the specific bariatric surgery procedure undergone by the participants.
4. **Night Eating Questionnaire (NEQ)**: The NEQ was employed as a primary diagnostic tool to identify the presence and severity of Night Eating Syndrome (NES) among participants. This questionnaire is a validated self-report instrument designed to capture the distinctive behaviors and patterns associated with NES. It comprises multiple items that ask respondents about their eating habits, specifically focusing on the timing and quantity of food consumption. Key aspects evaluated by the NEQ include the proportion of daily caloric intake consumed after the evening meal, incidents of nocturnal eating (defined as waking during the night to eat), mood fluctuations related to eating times, and sleep disturbances influenced by eating patterns. A critical threshold, a global NEQ score of 25 or higher, was adopted based on established diagnostic criteria to identify individuals likely to have NES.

### Statistical analyses

Data analysis was performed using SPSS software, version 20 (IBM, Armonk, New York, USA). Descriptive statistics were applied to summarize mean, standard deviation, and to provide frequency counts and percentages for categorical variables. The chi-squared test was utilized for examining associations among categorical variables, and the Mann–Whitney U test for analyzing continuous data. Spearman’s test was used for correlation analysis. Multivariate logistic regression analysis was carried out to identify factors independently associated with NES among participants. This analysis incorporated variables selected for their potential relevance to NES, with odds ratios calculated at a 95% confidence interval. A *P* value of less than 0.05 was considered statistically significant for all tests.

### Ethical considerations

This study received approval from the Research Ethics Committee of the Unit of Biomedical Ethics at the Faculty of Medicine, King Abdulaziz University, Jeddah, Saudi Arabia (Approval Number: 322 − 22). Prior to participation, all individuals gave their written informed consent, adhering to the ethical guidelines and ensuring the confidentiality and anonymity of their responses.

## Results

### Participant characteristics

In our assessment of 371 individuals, 154 patients were identified as meeting the study’s inclusion criteria. The demographic breakdown revealed 83 patients (53.9%) were female, and 130 patients (84.4%) were of Saudi nationality. The mean age of participants was 38.8 ± 11.4 years. Prior to undergoing bariatric procedures, the average weight of these individuals was 125.1 ± 29.0 kg, which significantly decreased to 80.5 ± 18.1 kg following the procedures. Correspondingly, the mean Body Mass Index (BMI) was reduced from 44.8 ± 8.2 kg/m² to 28.9 ± 5.8 kg/m². Sleeve gastrectomy was the predominant bariatric procedure, conducted on 131 patients (85.1%). Chronic diseases were reported by 109 patients (70.8%), with hypertension being the most commonly reported condition by 44 patients (28.8%). Furthermore, 41 patients (26.6%) reported adherence to a post-procedure diet (Table 1).

**Table 1:** Demographic and clinical characteristics of post-bariatric surgery patients evaluated for Night-Eating Syndrome (*N* = 154)

Variable		Frequency (%) or Mean ± SD
Age (years)		38.8 ± 11.4
Sex	Female	83 (53.9%)
	Male	71 (46.1%)
Nationality	Non-Saudi	24 (15.6%)
	Saudi	130 (84.4%)
Presence of chronic disease	Yes	45 (29.2%)
	No	109 (70.8%)
Specific chronic conditions	Diabetes mellitus	7 (15.5% of those with any disease)
	Dyslipidemia	5 (11.1% of those with any disease)
	Hypertension	13 (28.8% of those with any disease)
Height (cm)		166.6 ± 9.6
Weight (kg)	Before bariatric procedure	125.1 ± 29.0
	After bariatric procedure	80.5 ± 18.1
BMI (kg/m²)	Before bariatric procedure	44.8 ± 8.2
	After bariatric procedure	28.9 ± 5.8
Bariatric procedure	Intragastric balloon	1 (0.6%)
	Gastric band	2 (1.3%)
	Gastric bypass	20 (13.0%)
	Sleeve gastrectomy	131 (85.1%)
Adherence to Postoperative Diet	Yes	41 (26.6%)
	No	113 (73.4%)

N: number of patients; BMI: body mass index. Measurements are presented as mean ± SD for continuous variables and frequency (percentage) for categorical variables

### Factors associated with night-eating syndrome

Of the participants, 52 (33.8%) scored 25 or higher on the NEQ, indicating the presence of NES. Analysis showed non-significant negative correlations between NEQ scores and age, sex, as well as pre- and post-procedure weights and BMIs (Table 2). Notably, the prevalence of NES was significantly higher among female patients, with 35 females (42.2%) affected, compared to males, where 41 (57.8%) were found to have NES (*P* = 0.017). Among chronic conditions, diabetes was significantly associated with NES (*P* = 0.025), unlike other conditions examined in this study (Table 3).

**Table 2:** Correlation between physical characteristics and Night Eating Questionnaire scores in post-bariatric surgery patients

Variable		Correlation coefficient (r)	*P* value
Height (cm)		-0.050	0.516
Weight (kg)	Before bariatric procedure	-0.060	0.439
	After bariatric procedure	-0.130	0.099
BMI (kg/m²)	Before bariatric procedure	-0.005	0.954
	After bariatric procedure	-0.080	0.306

BMI: body mass index. Correlations were calculated using Spearman’s rank correlation coefficient. *P* values below 0.05 denote statistical significance

**Table 3:** Characteristics of patients with and without Night-Eating Syndrome in post-bariatric procedure patients

Variable		NES	No NES	*P* value
Age		40.4 ± 10.5	38.0 ± 11.9	0.140
Sex	Female	35 (42.2%)	48 (57.8%)	**0.017**
	Male	17 (23.9%)	54 (76.1%)	
Nationality	Non-Saudi	11 (45.8%)	13 (54.2%)	0.174
	Saudi	41 (31.5%)	89 (68.5%)	
Height (cm)	Height (cm)	165.0 ± 9.73	167.4 ± 9.5	0.123
Weight (kg)	Before procedure	120.7 ± 25.7	127.3 ± 30.5	0.618
	After procedure	78.9 ± 18.5	81.3 ± 18.0	0.417
BMI (kg/m²)	Before procedure	44.3 ± 8.5	45.1 ± 8.0	0.909
	After procedure	28.9 ± 6.2	28.9 ± 5.7	0.945
Presence of chronic disease	No	25 (55.6%)	20 (44.4%)	0.072
	Yes	77 (70.6%)	32 (29.4%)	
Specific chronic conditions	Diabetes mellitus	6 (85.7%)	1 (14.3%)	**0.025**
	Dyslipidemia	2 (40%)	3 (60%)	
	Hypertension	5 (38.5%)	8 (61.5%)	
	Other	6 (30%)	14 (70%)	
Adherence to postoperative diet	No	38 (33.6%)	75 (66.4%)	0.952
	Yes	14 (34.1%)	27 (65.9%)	

NES: Night-Eating Syndrome; BMI: body mass index. Data are presented as mean ± SD for continuous variables and frequency (percentage) for categorical variables. Continuous variables were analyzed using the Mann-Whitney U test and categorical variables using the Chi-Square test to compare differences and assess associations between groups with and without Night-Eating Syndrome. *P* values below 0.05 denote statistical significance

### Multivariable regression analysis model

The logistic regression analysis identified significant determinants of NES (Table 4). Being female was associated with an elevated risk of NES (OR: 2.33; 95% CI: 1.05–5.19; *P* = 0.030), and the presence of chronic diseases significantly increased the risk of NES (OR: 32.95; 95% CI: 2.33–464.44; *P* = 0.010). Specifically, diabetes mellitus was associated with a higher prevalence of NES (OR: 2.63; 95% CI: 1.23–5.88; *P* = 0.013).

**Table 4:** Determinants of Night Eating Syndrome in post-bariatric procedure patients: a logistic regression analysis

Variable	OR	95% CI	P value
Age at surgery (years)	1.01	(0.97–1.04)	0.576
Pre-surgery BMI (kg/m²)	1.00	(0.95–1.06)	0.770
Post-surgery BMI (kg/m²)	0.98	(0.92–1.05)	0.873
Gender (Female vs. Male)	2.33	(1.05–5.19)	0.030
Nationality (Saudi vs. Non-Saudi)	0.44	(0.16–1.18)	0.104
Type of bariatric procedure	2.61	(0.79–8.62)	0.115
Presence of chronic diseases	32.95	(2.33–64.44)	0.010
Specific chronic disease (Diabetes vs. Others)	2.63	(1.23–5.88)	0.013
Adherence to postoperative diet	1.04	(0.44–2.46)	0.918

*Abbreviations* OR: Odds Ratio; CI: Confidence Interval; BMI: Body Mass Index. Odds ratios greater than 1 suggest an increased probability of the Night Eating Syndrome. The analysis considers adjustments for all variables presented in the table. P-values below 0.05 denote statistical significance

## Discussion

This study aimed to evaluate the prevalence of NES in patients who had undergone bariatric surgery at King Abdulaziz University Hospital. Our findings shed light on the prevalence rates and associated factors of NES in this specific patient population and contribute to the growing body of knowledge on eating disorders post-bariatric surgery.

According to the NEQ, our study identified that 33.8% of patients in our sample met the criteria for NES. This prevalence rate aligns with previous research findings in the field. For instance, a study conducted in the United States found that 25% of overweight and obese patients exhibited NES symptoms after bariatric surgery [15]. However, variations in NES prevalence across studies may be attributed to differences in sample sizes and diagnostic criteria used for NES assessment.

Our study further delved into the demographic characteristics of patients with NES. The mean age of patients with NES in our study was 38.8 years, which is in line with similar studies that have reported relatively young ages among NES-affected individuals [18, 19]. This finding aligns with epidemiologic data indicating that NES tends to be less frequent among individuals over 65 years of age [11].

An intriguing finding in our study was the significantly higher prevalence of NES among female patients compared to male patients. This observation echoes the outcomes of previous investigations [15, 20] and reflects the well-established fact that women are more prone to developing eating disorders [21].

A notable finding in our study was the association between NES and chronic illnesses, particularly diabetes. A previous study conducted in the United States on patients with obesity and type 2 diabetes also identified NES as the most prevalent eating disorder in this population [22]. This reinforces the relationship between NES and metabolic conditions, as well as the impact of NES on dietary adherence and mood [23]. Furthermore, our study revealed that the majority of patients with NES underwent sleeve gastrectomy (90.3%). However, we found no significant relationship between NES prevalence and the type of bariatric procedure.

While our study explored various factors, including age, nationality, diet adherence, BMI, and type of bariatric procedure, we did not find any significant associations between these factors and the presence of NES. These results corroborate the findings of previous studies [24, 25]. Our study suggests that NES prevalence among post-bariatric surgery patients may not be strongly influenced by these demographic or clinical factors.

While our study offers valuable insights, it is essential to acknowledge its limitations. As a cross-sectional study, it is susceptible to recall bias. Moreover, the reliance on self-reported data through a questionnaire may introduce inaccuracies. To address these limitations, future research in this area should consider longitudinal designs and incorporate clinical assessments to provide a more comprehensive understanding of NES in post-bariatric surgery patients.

## Conclusion

In conclusion, our study contributes to the understanding of NES prevalence and risk factors among patients who have undergone bariatric surgery. The complex relationship between NES, demographic factors, and clinical variables necessitates further research to elucidate its underlying mechanisms and develop targeted interventions. Recognizing NES as part of postoperative care is essential for optimizing long-term outcomes and quality of life for this patient population. Moreover, our findings underscore the importance of continued research in the evolving field of bariatric surgery outcomes and eating disorders.

## Data Availability

The datasets generated and/or analyzed during the current study are available from the corresponding author upon reasonable request.

## References

[1] Welbourn R, Hollyman M, Kinsman R (2019). Bariatric surgery worldwide: baseline demographic description and one-year outcomes from the fourth ifso global registry report 2018. Obes Surg.

[2] Cohn I, Raman J, Sui Z (2019). Patient motivations and expectations prior to bariatric surgery: a qualitative systematic review. Obes Rev.

[3] Fraszczyk E, Luijten M, Spijkerman AMW (2020). The effects of bariatric surgery on clinical profile, DNA methylation, and ageing in severely obese patients. Clin Epigenetics.

[4] Conceicao EM, Utzinger LM, Pisetsky EM (2015). Eating disorders and problematic eating behaviours before and after bariatric surgery: characterization, assessment and association with treatment outcomes. Eur Eat Disord Rev.

[5] Crowley NM, LePage ML, Goldman RL, O’Neil PM, Borckardt JJ, Byrne TK (2012). The food craving questionnaire-trait in a bariatric surgery seeking population and ability to predict post-surgery weight loss at six months. Eat Behav.

[6] Taba JV, Suzuki MO, Nascimento FSD, et al. The development of feeding and eating disorders after bariatric surgery: a systematic review and meta-analysis. Nutrients. 2021;13. 10.3390/nu13072396. 10.3390/nu13072396 PMC8308796 34371904

[7] Stunkard AJ, Grace WJ, Wolff HG (1955). The night-eating syndrome; a pattern of food intake among certain obese patients. Am J Med.

[8] Shoar S, Naderan M, Mahmoodzadeh H, Shoar N, Lotfi D (2019). Night eating syndrome: a psychiatric disease, a sleep disorder, a delayed circadian eating rhythm, and/or a metabolic condition?. Expert Rev Endocrinol Metab.

[9] Allison KC, Lundgren JD, O’Reardon JP (2010). Proposed diagnostic criteria for night eating syndrome. Int J Eat Disord.

[10] Tozzi F, Thornton LM, Klump KL (2005). Symptom fluctuation in eating disorders: correlates of diagnostic crossover. Am J Psychiatry.

[11] Rand CS, Macgregor AM, Stunkard AJ (1997). The night eating syndrome in the general population and among postoperative obesity surgery patients. Int J Eat Disord.

[12] Nasirzadeh Y, Kantarovich K, Wnuk S (2018). Binge eating, loss of control over eating, emotional eating, and night eating after bariatric surgery: results from the toronto bari-psych cohort study. Obes Surg.

[13] Unal S, Sevincer GM, Maner AF (2019). [prediction of weight regain after bariatric surgery by night eating, emotional eating, eating concerns, depression and demographic characteristics]. Turk Psikiyatri Derg.

[14] Ivezaj V, Lawson JL, Lydecker JA, Duffy AJ, Grilo CM (2022). Examination of night eating and loss-of-control eating following bariatric surgery. Eat Weight Disord.

[15] Brancati GE, Barbuti M, Calderone A (2022). Prevalence and psychiatric comorbidities of night-eating behavior in obese bariatric patients: preliminary evidence for a connection between night-eating and bipolar spectrum disorders. Eat Weight Disord.

[16] Al Khalifa K, Al Ansari A (2018). Quality of life, food tolerance, and eating disorder behavior after laparoscopic gastric banding and sleeve gastrectomy - results from a middle eastern center of excellence. BMC Obes.

[17] Allison KC, Wadden TA, Sarwer DB (2006). Night eating syndrome and binge eating disorder among persons seeking bariatric surgery: prevalence and related features. Obes (Silver Spring).

[18] Nolan LJ, Geliebter A (2016). Food addiction is associated with night eating severity. Appetite.

[19] Vinai P, Ferri R, Anelli M (2015). New data on psychological traits and sleep profiles of patients affected by nocturnal eating. Sleep Med.

[20] Petroni ML, Barbanti FA, Bonadonna R (2019). Dysfunctional eating in type 2 diabetes mellitus: a multicenter Italian study of socio-demographic and clinical associations. Nutr Metab Cardiovasc Dis.

[21] Qian J, Wu Y, Liu F (2022). An update on the prevalence of eating disorders in the general population: a systematic review and meta-analysis. Eat Weight Disord.

[22] Gonzalez JS, Hood KK, Esbitt SA, Mukherji S, Kane NS, Jacobson A. Psychiatric and psychosocial issues among individuals living with diabetes. Diabetes in America 3rd edition. 2018. 33651537

[23] Allison KC, Crow SJ, Reeves RR (2007). Binge eating disorder and night eating syndrome in adults with type 2 diabetes. Obes (Silver Spring).

[24] Silen Y, Keski-Rahkonen A (2022). Worldwide prevalence of dsm-5 eating disorders among young people. Curr Opin Psychiatry.

[25] Ozsoy Z, Demir E (2018). Which bariatric procedure is the most popular in the world? A bibliometric comparison. Obes Surg.

